# The role of Qa-2, the functional homolog of HLA-G, in a Behcet's disease-like mouse model induced by the herpes virus simplex

**DOI:** 10.1186/1476-9255-7-31

**Published:** 2010-06-24

**Authors:** Meeyoung Lee, Bunsoon Choi, Hyuk Jae Kwon, Ju A Shim, Kyung Sook Park, Eun-So Lee, Seonghyang Sohn

**Affiliations:** 1 Laboratory of Cell Biology, Ajou University Institute for Medical Sciences, Suwon, Korea; 2 Department of Biology, Sungshin University, Seoul, Korea; 3 Department of Dermatology, Ajou University School of Medicine, Suwon, Korea; 4 Brain Korea 21 Project for Medical Science, Ajou University, Suwon, Korea

## Abstract

**Background:**

It has been suggested that the HLA-G molecule is a genetic risk factor for Behcet's disease (BD). In this study, we evaluated the level of Qa-2, a murine nonclassical class I MHC molecule and possible functional homolog of HLA-G, to determine if it was associated with various symptoms of BD-like mice. In addition, we investigated siRNA (small interfering RNA) treatment to determine if it inhibited Qa-2 expression, thereby changing the symptoms of mice.

**Methods:**

RNA interference (RNAi) and vector transfection were employed to manipulate gene expression *in vivo *in mice. siRNA (small interfering RNA) or Qa-2 expression vector was applied to inhibit or up-regulate Qa-2 expression, respectively.

**Results:**

The Qa-2 levels in granulocytes were lower in BD-like mice than in normal controls. The silencing of Qa-2 by intravenous injection of siRNA (500 nmol/mouse, 4 times at 3-day intervals) specifically reduced the Qa-2 levels and worsened the BD-like symptoms.

**Conclusions:**

Silencing Qa-2 by injecting siRNA into mice resulted in deterioration of symptoms in BD-like mice.

## Background

Since HLA-G (human leukocyte antigen-G) was first detected by Geraghty et al. [1], it has been reported that HLA-G protein is expressed at the feto-maternal interface during pregnancy [2] and on a subset of thymic epithelial cells [3], and that it is also involved in maintenance of tolerance of the maternal immune system toward the semi-allogeneic fetus. HLA-G is also expressed in other tissues such as intestinal mucosa [4] and PBMC [5]. Numerous studies have evaluated the relevance of HLA-G under pathologic conditions such as transplantation, autoimmunity, cancer, and hematologic malignancies [6]. HLA-G interacts with different natural killer (NK) cell receptors and is able to inhibit NK and T-cell cytotoxicity, as well as T-cell proliferation [7]. Interestingly, HLA-G has been described as a unique ligand of the killer cell inhibitory receptor, KIR2DL4, which is expressed on the surface of all NK cells [8]. Furthermore, HLA-G inhibits the transendothelial migration of NK cells [9], shifts the cytokine balance toward Th2 dominance [10], and suppresses the proliferation of allogeneic CD4^+ ^T lymphocytes [11,12]. Taken together, HLA-G exerts specific inhibitory effects against immune cells. In addition, recent studies indicate unexpected expression of HLA-G proteins in chronic cutaneous inflammatory diseases, such as psoriasis [13] and atopic dermatitis [14].

Behcet's disease (BD) is a chronic multi-systemic disorder that involves the gastrointestinal, mucocutaneous, ocular, vascular, central nervous, and articular systems. BD has a chronic course that includes periodic exacerbations and progressive deterioration [15]. Although the etiology of BD is unclear, viral infection has long been postulated as one of its main factors. The viral hypothesis has been verified by detection of the virus in saliva [16], intestinal ulcers [17], and genital ulcers [18] of patients with BD since it was first proposed by Hulûsi Behçet [19]. Furthermore, inoculation of the earlobe of ICR mice with herpes simplex virus (HSV) enables development of a BD-like animal model [20]. Manifestations in mice following HSV inoculation involve multiple symptoms such as oral ulcers, genital ulcers, skin ulcers, eye symptoms, gastrointestinal ulcers, arthritis, and neural involvement, as well as skin crusting. The frequency of these symptoms is similar to that of patients with BD [21]. In addition to viral causes of BD, several studies have identified lymphocyte dysfunction as a possible cause [22,23]. Thus, attention has been focused on the T helper (Th) 1 and Th2 cytokines, with Th1 cells perhaps playing a more important role in the immunopathogenesis of BD [24]. When the Th2 adjuvant, aluminium hydroxide (alum), was mixed with ovalbumin (OVA) and injected into mice suffering from BD, their cutaneous symptoms were improved [25].

Park et al. [26] reported that the frequency of haplotypes containing a HLA-G *3741_3754 *14 base pair insertion and 1597*delC was increased in BD patients. Moreover, individuals who were homozygous with the 3741_3754*ins14/*ins14 genotype were found to have a risk of BD that was 2.7-times greater than that of the controls. The HLA-G 3741*+14bp induces a significantly lower expression level than the complete HLA-G mRNA isoforms. In addition, the HLA-G *3741_3754 *14-base pair insertion allele was found to occur significantly more frequently in BD patients with ocular, arthritis, and CNS symptoms than in controls, and this insertion was found to be related to the lower serum level of HLA-G [26]. The authors who presented these findings suggested that these HLA-G allelic variants are genetic risk factors for BD. In addition, the HLA-G*010101 alleles have been shown to have a significantly lower frequency in BD patients than in control subjects [27].

As a result, it is important to determine if HLA-G contributes to the pathogenesis of BD. To accomplish this, Qa-2 expression, the functional homolog of HLA-G in mice, was identified and modulated by small interfering RNA (siRNA) and the Qa-2 expression vector. The results of this study confirmed that decreased Qa-2 levels are related to changes in the disease pattern and deterioration of BD-like symptoms.

## Methods

### Animals, induction of BD-like symptoms, and scoring of BD activity

Five-week-old ICR male mice were used in this study. To induce a BD-like disease in mice, their earlobes were scratched with a needle and then inoculated with 1.0 × 10^6 ^plaque forming units/ml of HSV type 1 (F strain). Virus inoculation was performed twice with a 10-day interval, after which the mice were observed for 30 weeks. Mice were housed in conventional temperature- and light-controlled rooms (20-22°C, 12 h light cycle starting at 8:00 a.m.) and had free access to food and water. During the experiment, the animals were observed closely. Mice were handled in accordance with the protocols approved by our institutional animal care committee. Manifestations in mice after HSV inoculation involved multiple symptoms including oral ulcers, genital ulcers, skin ulcers, eye symptoms, intestinal ulcers, arthritis, and neural involvement, as well as skin crusting. Oral, genital, and other skin ulcers (including bulla and crust), and eye symptoms were all classified as major symptoms, while other symptoms were classified as minor symptoms [20]. Overall, 15% of the HSV-injected mice developed BD-like symptoms. The disappearance of symptoms and decrease in lesion size constituted an improvement, similar to in human patients.

The animals were observed once a week after HSV inoculation, at which time the severity of BD was determined according to the BD activity index, as outlined in the Behcet's Disease Current Activity Form 2006 http://www.behcet.ws/pdf/BehcetsDiseaseActivityForm.pdf. The occurrence of the following symptoms in the mouse model were selected for analysis: mouth ulceration, genital ulceration, erythema, skin pustules, skin ulceration, joints-arthritis, diarrhea, red eye (right, left), reduced vision (right, left), loss of balance, discoloration, and swelling of the face. The score of each symptom was one, and the total score before and after treatment was used to determine the severity of BD. Mice exhibiting significantly reduced symptoms were photographed to document improvement after treatment.

### Synthesis and in vitro test of siRNA

Qa-2 siRNA oligonucleotides with the following sense and anti-sense sequences were designed and synthesized by Dharmacon (Chicago, IL, USA). The Qa-2 protein was encoded by four genes in the Q region, Q6, Q7, Q8 and Q9. These genes have a typical class I MHC gene structure involving exon 1 (leader peptide), exon 2 (α1 domain), exon 3 (α2 domain), exon 4 (α3 domain), exon 5 (transmembrane domain), and exons 6, 7 and 8 (cytoplasmic domains). As shown in Table 1, we selected four sequences located in each domain to synthesize siRNA. To confirm the function of interference, the synthesized siRNA was tested *in vitro *in peripheral blood mononuclear cells (PBMC). To accomplish this, PBMCs were isolated from 5-6 week-old ICR mice and cultured at 1 × 10^5 ^cells/ml in DMEM medium with 1% antibiotics and 10% FBS. siRNA (200 nM) was incubated with 3 μL of oligofectamin (Gibco-Invitrogen, Rockville, MD) in 200 μL of DMEM medium. After 24 h of treatment with siRNA, the PBMCs were harvested and subjected to RT-PCR.

**Table 1:** Qa-2 siRNA oligonucleotide sequences

Qa-2 domain	siRNA oligonucleotides sequences
Leader peptide domain	5'-CAACACUCGCAAUAUU-3'(sense) 3'-GUUGUGAGCGACGUUAUAA-5'(antisense)

α3 domain	5'-AGGUCUUAUGGUGCUGUCAUU-3'(sense) 3'-UUUCCAGAAUACCACGACAGU-5'(antisense)

Transmembrane domain	5'-UGUGAUGAAUAGGAGGUGAUU-3'(sense) 3'-UUACACUACUUAUCCUCCACU-5'(antisense)

Cytoplasmic membrane domain	5'-UAGAGCUCUGAUAGAUCUCUU-3'(sense) 3'-UUAUCUCGAGACUAUCUAGAG-5'(antisense)

### In vivo siRNA injection

For application to mice, 500 nM of siRNA in 200 μL of 5% glucose, including transfection reagent jetPEI (Polyplus, France, Illkirchcedex), was intravenously injected into mice one to four times with a three day interval between injections. Two-days after the last injection, mice were photographed and the PBMCs were analyzed using a fluorescence-activated cell sorter (FACS). The control group was injected with 200 μL of 5% glucose. Qa-2 leader peptide domain siRNA did not down-regulate the Qa-2 mRNA level in *in vitro *PBMC cultures when compared to other domains; therefore, the leader peptide domain siRNA was injected as a control. For *in vivo *administration to mice, 1.5 μL of transfection reagent was mixed with 5% glucose and siRNA. The Qa-2 siRNA was mixed with α3 domain, transmembrane domain and cytoplasmic domain in equal amounts, after which it was administered to mice.

### Flow cytometry

To analyze the Qa-2 expression, cells were harvested and fixed with 4% formaldehyde in 1% fetal bovine serum containing PBS for 20 min at room temperature, after which they were incubated with FITC-conjugated anti-Qa-2 antibody (eBioscience, San Diego, CA, USA). Stained cells were analyzed in FACS Vantage using the Cell Quest software (Becton Dickinson, Franklin Lakes, NJ, USA) by collecting at least 10,000 gated lymphocytes [7].

### Reverse transcription PCR (RT-PCR)

Total RNA was isolated using TRIzol (Life Technologies, Helgerman, CT) according to the manufacturer's recommendations. Two μg of total RNA were used as a template for cDNA synthesis, which was conducted using a SuperScript III First-Strand Synthesis System for RT-PCR kit (Invitrogen, Carlsbad, CA). The cDNA was amplified by PCR using the following primers: Qa-2, Sense: 5' - AGGTCTTAT GGTGCTGTCAC-3', Anti sense: 5'- TGTGTAATTCTGCTCCTTCC -3'; β-actin, Sense: 5'-TGGAATCCTGTGGCATCCATGAAAC -3', Antisense: 5'-TAAAACGCAGCTCAGTAACAGTCCG-3'; IFNγ, Sense: 5'-AGCGGCTGACTGAA CTCAGATTGTAGCTTGTACCTTTACTTCACTG-3', Antisense: 5'-GTCACAGTTTTCA GCTGTATAGGG-3'. Amplified PCR products were visualized on 1.2% agarose gels.

### Real Time PCR

For real-time SYBR Green RT-PCR, a 20-μl reaction containing 10 μl of 2× Quantitect SYBR Green Master Mix (Qiagen, Valencia, CA, USA) was employed. The master mix was composed of hot start Taq polymerase, a 0.4 μL mix of 2 reverse transcriptases, 0.5 μL (10 ng/μL) of template and 0.8 μL of primers. An ABI 7900 HT thermal cycler (Lab Centraal B.V., Haarlem, The Netherlands) was used for all real-time RT-PCR assays. Reverse transcription was conducted at 50°C for 30 min, followed by denaturation at 95°C for 15 min. DNA was amplified by subjecting the samples to 40 cycles of 95°C (30 s), 55°C (30 s), and 72°C (30 s). Real-time RT-PCR data were collected for 15 sec at 75°C to avoid non-specific fluorescence due to the formation of primer dimers at low template concentrations. For generation of standard quantitation curves, the cycle threshold values were plotted proportionally against the logarithm of the input copy numbers. Negative controls were included in each run.

### Qa-2 vector construction

Qa-2 cDNA was amplified from total RNA extracted from ICR mice lymph nodes by reverse transcriptase - polymerase chain reaction (RT-PCR) using the following primers: sense 5'-CGGGATCCCGATGGCTCTAACAATGCTGC-3', antisense 5'-CGGAATTCCGCTTCGTGTGAAAGTATGGAG-3'. The sense primer included the BamH1 restriction site and the antisense primer included the EcoR1 restriction site. The cDNA was subsequently digested with *Bam*HI and *Eco*RI and then inserted into eukaryotic expression vector pcDNA3.1 (Invitrogen, Carlsbad, CA, USA). Verification of the recombinant construct was performed by DNA sequencing. The empty vector pcDNA3.1 was used as a control. All plasmids were purified by two rounds of passage through Endo-Free columns (Qiagen, Chatsworth, CA, USA), as described elsewhere [28].

### Qa-2 vector transfection to HeLa cells

HeLa cells were maintained in Dulbecco's modified Eagle medium (DMEM) supplemented with 2 mM glutamine, 100 units/ml penicillin, 100 μg/ml streptomycin, and 5% (v/v) dextran-charcoal-treated fetal bovine serum at 37°C in 5% CO_2_. Cells were plated at 10^6 ^cells/10 cm dish the day before transfection, after which they were transfected using a lipofectimine kit (Invitrogen, Paisley, UK) according to the manufacturer's instructions. The vector pcDNA3.1 was transfected into HeLa cells as a control.

### Administration of Qa-2 vector to mice

Normal and BD mice were intraperitoneally injected once with 50 ng of pcDNA 3.1 or pcDNA 3.1 Qa-2 vector per mouse, and their splenocytes or macrophages were isolated three days later and analyzed by flow cytometry. Vector mixed with transfection reagent jetPEI was injected into mice and the frequency of Qa-2 protein expression was analyzed by FACS.

### Statistical analysis

All data are presented as the mean ± SE. Statistical differences between groups were determined using a Student's *t *test and the Bonferroni correction. Statistical analysis was conducted using MedCalc^® ^version 9.3.0.0.

## Results

### Qa-2 mRNA and Qa-2 positive PBMCs were lower in BD symptomatic mice than in normal healthy mice

**Figure 1:**
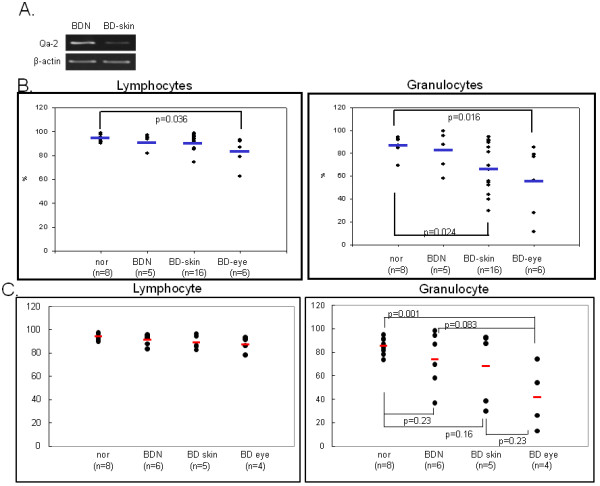
**Qa-2 expression in PBMC of BD**. A. RT-PCR demonstrated that mRNA expression was lower in PBMC of BD skin than in BD normal mice. B. The frequency of Qa-2 in PBMC of normal healthy controls, BD asymptomatic (BD normal) mice, BD mucocutaneous symptomatic mice (BD skin), and BD mucocutaneous and ocular symptomatic mice (BD eye) as determined by FACS analysis. In lymphocytes, the Qa-2 levels in BD eye mice were significantly lower than in normal healthy mice (p = 0.036). These levels were also lower than in BD skin mice, although this difference was not significant. In granulocytes, the Qa-2 levels in BD eye mice were significantly lower than in normal healthy mice (p = 0.016). The Qa-2 levels in BD eye mice were lower than in normal and BD skin mice, although this difference was not statistically significant. Qa-2 levels in BD skin were significantly lower than in normal controls (p = 0.024). C. The portion of Qa-2 positive cells in lymphocytes or granulocytes. The frequency of Qa-2 positive cells in the granulocytes of BD skin and BD eye mice was lower than in normal controls and BD normal mice (BDN). The frequencies of Qa-2 positive cells in BD eye mice were significantly lower than those in normal controls (p = 0.001).

RT-PCR revealed that Qa-2 mRNA expression in peripheral blood mononuclear cells (PBMC) of mucocutaneous symptomatic BD mice was down-regulated when compared to asymptomatic BD mice, despite HSV inoculation (BD normal, BDN) (Figure 1A). Next, Qa-2 levels in PBMCs obtained from normal healthy mice, BD asymptomatic mice (BDN), BD skin symptomatic mice (BD skin), and BD eye symptomatic mice (BD eye) were analyzed by flow cytometry. The symptoms of BD skin consisted of typical mucocutaneous symptoms in mice without ocular symptoms, while those of BD eye mice consisted of ocular symptoms with mucocutaneous symptoms. After FACS staining, lymphocytes and granulocytes were separated by gating. In lymphocytes, Qa-2 positive cells accounted for 94.78 ± 3.56% in normal healthy mice, 92.56 ± 6.13% in BD normal mice, 91.73 ± 5.96% in BD skin, and 84.49 ± 11.95% in BD eye mice. BD eye mice were found to have a statistically lower number of Qa-2 positive cells than normal healthy mice (p = 0.036). In granulocytes, Qa-2 positive cells were 87.01 ± 7.97% in normal healthy mice, 82.29 ± 17.47% in BD normal mice, 67.9 ± 21.42% in BD skin mice, and 56.00 ± 30.49% in BD eye mice. BD skin and BD eye mice showed significantly lower levels of Qa-2 positive cells than normal healthy mice (p = 0.024, p = 0.016 each) (Figure 1B). The portion of Qa-2 positive cells in the granulocytes of BD skin and BD eye mice was lower than that of normal control and BD normal (BDN) mice. The portion of Qa-2 positive cells in the granulocytes of BD eye mice was significantly lower than that of normal controls (p = 0.001) (Figure 1C). As shown in Figure 1, the decreased level of Qa-2 was related to the BD symptoms.

### RNA interference of Qa-2 transcription *in vitro*; Qa-2 siRNA reduced Qa-2 mRNA levels in PBMCs of normal mice

**Figure 2:**
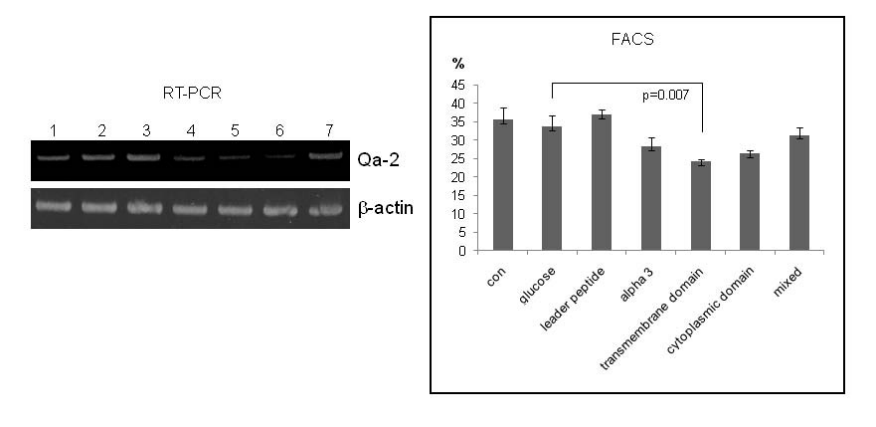
**Qa-2 siRNA reduced Qa-2 mRNA and the frequency of Qa-2 positive cells in PBMC of normal mice**. PBMC isolated from mice were transfected with Qa-2 siRNAs with different Qa-2 domains for 24 hrs, and the expression of Qa-2 was then determined by reverse transcriptase-PCR and FACS analysis. Lanes 4, 5 and 6 (α3 domain, transmembrane domain, and cytoplasmic domain, respectively) showed that siRNA effectively reduced the Qa-2 mRNA levels. Lane 3 (leader peptide) did not decrease the Qa-2 level. Lane 7 (a mixture of leader peptide, α3 domain, transmembrane domain, and cytoplasmic domain) also did not decrease the Qa-2 level. Lane 1, control (not treated); Lane 2, 5% glucose treated; Lane 3, leader peptide 200 nmole; Lane 4, α3 domain 200 nmole; Lane 5, transmembrane domain 200 nmole; Lane 6, cytoplasmic domain 200 nmole; Lane 7, mixed 200 nmole (leader peptide + α3 domain+ transmembrane domain + cytoplasmic domain).

PBMCs isolated from normal mice were transfected for 24 h with Qa-2 siRNA with different domains, after which the expression of Qa-2 was determined by reverse transcriptase-PCR. siRNA for the α3 domain, transmembrane domain, and cytoplasmic domain inhibited the Qa-2 level; however, the leader peptide domain did not. Mixed siRNA consisting of equal amounts each of these four domains did not downregulate the Qa-2 mRNA level. Flow cytometric analysis also showed a decreased frequency of Qa-2 expression in the Qa-2 siRNA domain-treated groups, except for the leader peptide domain (Figure 2).

### Downregulation of Qa-2 by intravenous injection of siRNA into BD mice

**Figure 3:**
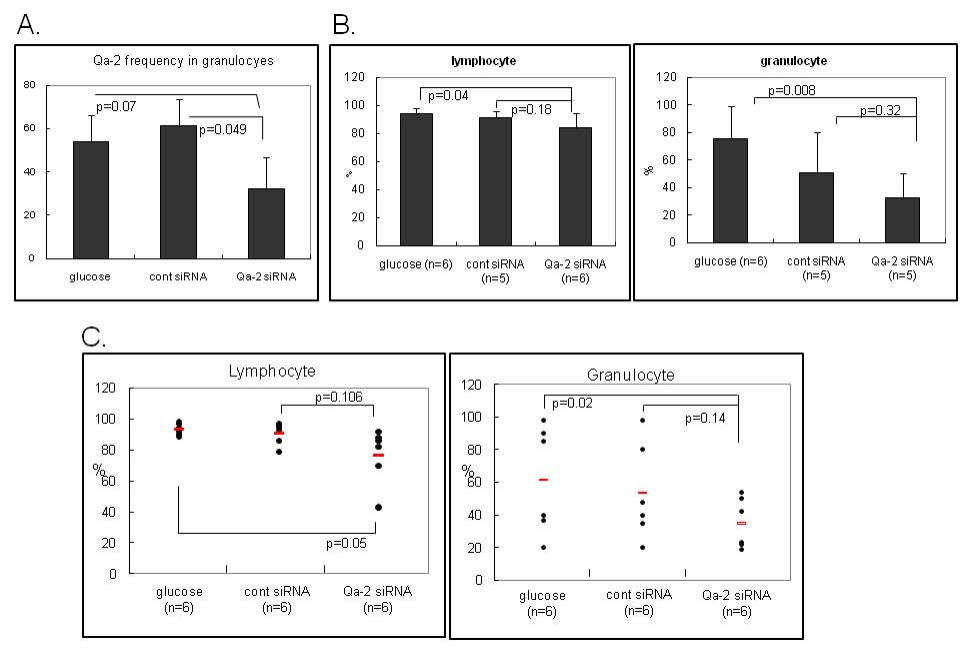
**Down-regulation of Qa-2 after intravenous injection of siRNA into BD mice**. A. BD mice were injected once with control siRNA or Qa-2 siRNA (500 nmol/mouse), which was composed of the α3 domain, transmembrane domain, and cytoplasmic domain. PBMC collected from the orbital sinus before and 1 day after injection were analyzed by flow cytometry. Qa-2 siRNA effectively reduced the Qa-2 levels in the PBMC of BD mice. B. Two days after injection, the frequency of Qa-2 positive lymphocytes and granulocytes was analyzed in Qa-2 siRNA injected BD mice. In lymphocytes and granulocytes, Qa-2 siRNA significantly reduced the number of Qa-2 positive cells when compared to glucose-injected control mice. C. The frequency of Qa-2 positive cells in mice that were injected with Qa-2 siRNA four times. Specifically, 500 nmol control siRNA or Qa-2 siRNA in 200 μl of 5% glucose solution was intraperitoneally injected four times with a three day interval between injections, and the PBMC were analyzed by FACS two days after the last injection. In lymphocytes and granulocytes, Qa-2 siRNA significantly reduced the Qa-2 positive cells when compared to glucose injected control mice.

Next, an siRNA mixture composed of the siRNA of the α3 domain, transmembrane domain and the cytoplasmic domain was injected into BD mice. Five to six individual BD mice in each group were intravenously injected once with 5% glucose or 500 nmol of Qa-2 siRNA or control siRNA, and their PBMCs were analyzed one day and two days later by flow cytometry. One day after Qa-2 siRNA injection, the number of Qa-2 positive granulocytes was 32.18 ± 14.64%, which was significantly lower (p = 0.049) than that of mice treated with 5% glucose (54.21 ± 1.89%) or leader peptide (61.32 ± 12.27%) (Figure 3A). In lymphocytes, the Qa-2 positive cell counts did not differ significantly among groups. Two days later, the frequency of Qa-2 positive cells was 84.12 ± 10.34% in Qa-2 siRNA injected mice, while it was 94.23 ± 3.86% of glucose injected control mice in lymphocytes (p = 0.029). In granulocytes, the frequency of Qa-2 positive cells was 42.18 ± 28.40% in Qa-2 siRNA injected mice, while it was 75.65 ± 23.59% in glucose injected control mice (p = 0.008). These findings demonstrated that Qa-2 siRNA effectively reduced the frequencies of Qa-2 positive cells in lymphocytes and granulocytes in BD mice (Figure 3B). To determine if repeated administration can reduce the Qa-2 level more efficiently, the frequency of Qa-2 positive cells in BD mice that were injected with Qa-2 siRNA four times was analyzed. To accomplish this, 500 nmol control siRNA or Qa-2 siRNA in 200 μl of 5% glucose solution was intraperitoneally injected four times with a three day interval in between injections. Two days after the last injection, the PBMCs were analyzed by FACS. In lymphocytes and granulocytes, Qa-2 siRNA led to a significant reduction in Qa-2 positive cells when compared to glucose injected control mice (p = 0.05, p = 0.02 each) (Figure 3C). However, the reduction in Qa-2 level observed in response to one and four injections did not differ significantly.

### The change in symptoms after Qa-2 siRNA injection into BD mice

**Figure 4:**
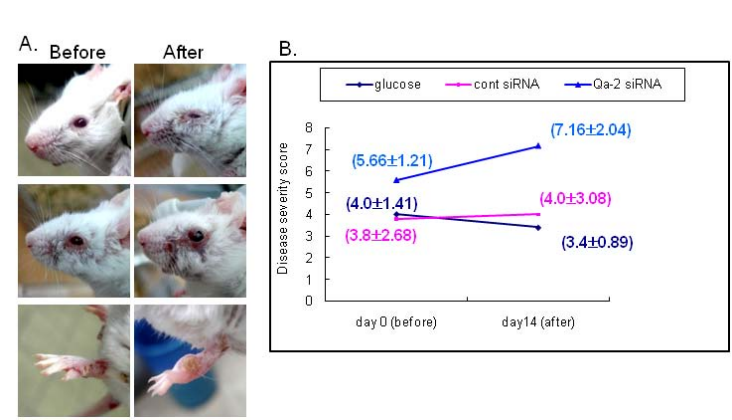
**Qa-2 siRNA deteriorated BD symptoms**. For each mouse, 500 nmol each of control siRNA or Qa-2 siRNA in 200 ml of 5% glucose solution was intraperitoneally injected four times with three day intervals, and the symptoms were photographed (A) and the severity score was analyzed (B) two days after the last injection. The severity was lower in Qa-2 siRNA injected BD mice when compared to control siRNA injected BD mice, although this change was not significant. The disease score was estimated according to the Patients Index Score, Behcet's disease current activity form 2006, ICBD. The symptoms of BD mice deteriorated after treatment with Qa-2 siRNA. Deterioration occurred in three of six BD mice (A). When treated with control siRNA, the deterioration occurred in one of six mice, while no change was observed in any of the mice injected with 5% glucose.

To determine if down-regulation of Qa-2 could influence the symptoms of BD, changes in symptoms (Table 2) and the disease severity score were examined after administration of siRNA to BD mice. Specifically, Qa-2 siRNA was intravenously injected into BD mice four times with a three day interval between treatments. After the injection of siRNA, deterioration occurred in three of six BD mice (Figure 4A). However, in mice treated with control siRNA, the deterioration only occurred in one of the six mice. In addition, there was no change in symptoms observed in any of the seven BD mice injected with 5% glucose. The change in symptoms was scored according to the severity score of BD, which is outlined in the BD Current Activity Form. As shown in Figure 4B, the score of the Qa-2 siRNA-injected group increased from 5.66 ± 1.21 to 7.16 ± 2.04, although this change was not statistically significant (p = 0.07). In contrast, the score in the control siRNA injected group increased to 4.0 ± 3.08 from 3.8 ± 2.68, while that of the glucose injected group changed from 4.0 ± 1.41 to 3.4 ± 0.89.

**Table 2:** Changes in symptoms after Qa-2 siRNA injection into BD mice

siRNA	Deteriorated number/ total number
Qa-2 siRNA	3/6

Leader peptide siRNA	1/6

Glucose	0/7

The symptoms of BD mice deteriorated following treatment with Qa-2 siRNA. Qa-2 siRNA was intravenously injected into BD mice four times with a three day interval between injections. Deterioration occurred in three of six BD mice.

### Qa-2 siRNA increased IFNγ mRNA levels in spleens of BD mice

**Figure 5:**
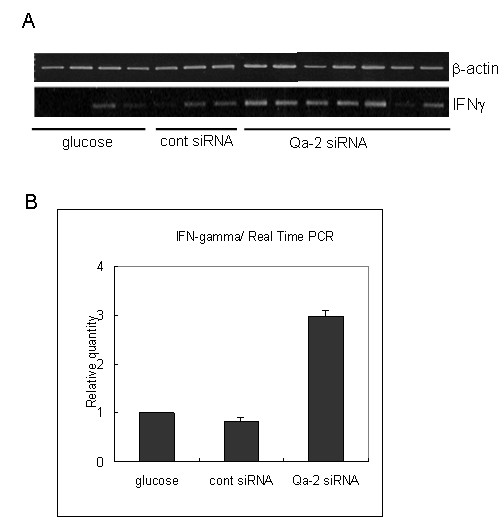
**The expression of IFN-γ mRNA in the spleens of glucose, control siRNA, and Qa-2 siRNA-injected BD mice**. The expression of IFN-γ mRNA as shown by RT-PCR (A) and real time PCR (B). IFNγ mRNA expression was increased in the Qa-2 siRNA-injected mice.

Recent *in vitro *studies have suggested that some duplex siRNA sequences have non-specific effects and can induce an IFN response, particularly at high concentrations [29,30]. However, further studies are needed to determine if these series of reactions can occur *in vivo *and if this can occur in response to our siRNA sequences [31]. Xie et al. reported that non-viral siRNA delivery to diseased tissue does not elicit an immune response [32]. To determine the IFNγ mRNA expression, the spleen tissues of BD mice that were injected with siRNA four times were subjected to reverse transcriptase PCR (RT-PCR) (Figure 5A) and real time PCR (Figure 5B). The IFNγ mRNA expressions were increased in the Qa-2 siRNA-injected mice when compared to the control siRNA or glucose injected group. Increased IFNγ was not due to siRNA, but rather to suppressed Qa-2 expression because control siRNA did not increase the level of IFNγ. These findings are in accordance with the finding that HLA-G-expressing cells showed significantly reduced levels of IFNγ [33].

### Qa-2 expression vector decreased the frequency of IFNγ stained macrophages in BD mice

**Figure 6:**
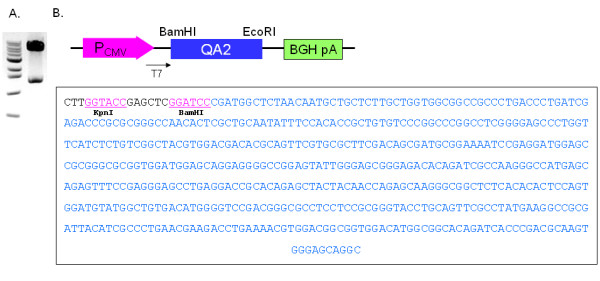
**Construction of a Qa-2 expression vector**. A. pcDNA3.1-Qa-2 was constructed by insertion of the full length mouse Qa-2 gene into the EcoR1 and BamH1 restriction site (expected size: 1.36 kb + 5.43 kb). The inserted Qa-2 gene was confirmed by digestion with EcoRI and BamHI. B. Vector inserted Qa-2 was sequenced using T7 promoter.

**Figure 7:**
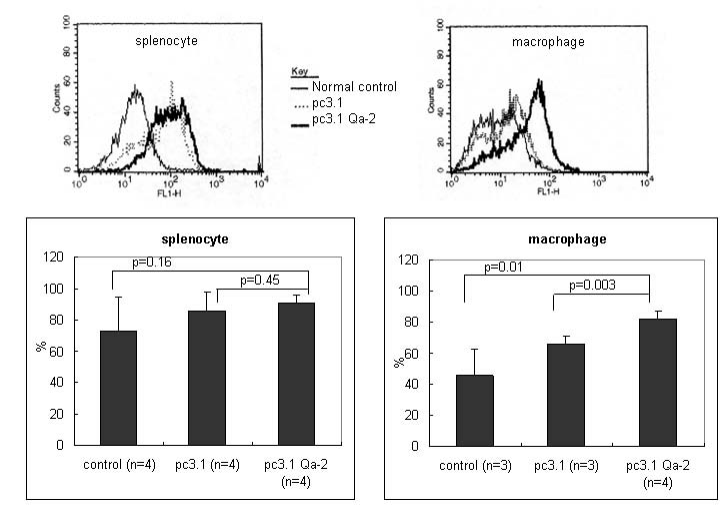
**Expression of pc3.1DNA Qa-2 vector *in vivo *in normal mice**. The frequency of Qa-2 protein in splenocytes and macrophages isolated from normal mice injected with 50 μg pc3.1DNA Qa-2 vector as determined by FACS analysis. The macrophages isolated from mice injected with pc3.1DNA Qa-2 vector showed a higher frequency of Qa-2 positive cells when compared to the non-injected control (p = 0.01) and pc3.1 DNA vector-injected mice (p = 0.003). The splenocytes also showed a higher frequency of Qa-2 positive cells when compared to the non-injected control and pc3.1 DNA vector injected mice, although this difference was not significant.

**Figure 8:**
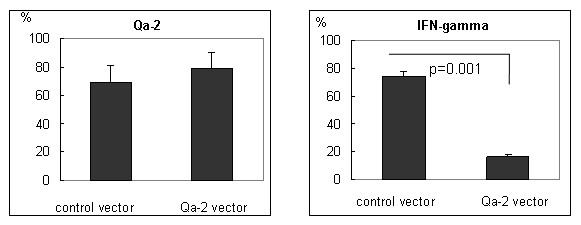
**Expression of Qa-2 and IFNγ in the macrophages of Qa-2 vector injected BD mice as shown by FACS analysis**. BD mice were intraperitoneally injected once with 50 μg of pcDNA 3.1 vector or pcDNA 3.1 Qa-2 vector and their macrophages were analyzed four days later by flow cytometry. Qa-2 positive cells were increased in the Qa-2 vector injected mice, whereas IFNγ positive cells decreased.

To confirm if Qa-2 could influence IFNγ expression, Qa-2 vector was constructed in PC3.1 vector and then administered to normal and BD mice. Cloning of the Qa-2 gene of pGEM-Qa-2 into pcDNA3.1 vector was confirmed by digestion with EcoRI and BamHI (Figure 6A), after which the inserted sequence was confirmed by sequencing using T7 promoter (Figure 6B). The vector was intraperitoneally injected once into mice, and peritoneal macrophages and splenocytes were isolated four days later. As shown in Figure 7, the frequency of Qa-2 expressing cells in splenocytes increased to 94.53 ± 0.64% in the Qa-2 vector injected mice, while it was 89.83 ± 2.66% in control vector injected mice (p = 0.45). Additionally, their frequency in macrophages increased to 82.25 ± 5.62% in the Qa-2 vector injected mice, while it was 67.53 ± 4.66% in control vector injected mice (p = 0.003). The IFNγ levels in macrophages of Qa-2 vector-injected mice also decreased to 16.60 ± 6.11%, while they were 66.24 ± 7.28% in control mice (p < 0.001) (Figure 8). Qa-2 expression vector appeared to work in macrophages, and these effects were accompanied by a decrease in IFNγ.

### The frequency of NK cells in BD and BDN mice

**Figure 9:**
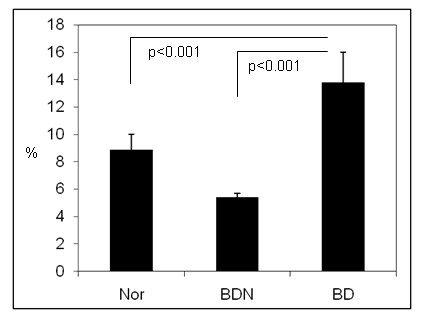
**The frequency of NK cells in BD and BDN mice**. The frequency of NK cells was analyzed in BD and BDN mice using flow cytometry.

To confirm the relationship between HLA-G and the NK cells, the frequency of NK cells was observed in BD and BDN mice using flow cytometry. As shown in Figure 9, the frequency of NK cells in splenocytes was 13.8 ± 2.2% in BD mice (n = 9) when compared to BDN mice (5.4 ± 0.3%) (n = 5, p < 0.001) and normal mice (8.9 ± 1.1%) (n = 7, p < 0.001). The frequency of NK cells in BD mice was higher than BDN. These findings indicate that down-regulation of HLA-G may influence the higher frequency of NK cells in BD mice.

## Discussion

In this study, Qa-2 expression in HSV-induced BD mice was investigated and compared to that of normal mice and BD asymptomatic mice. The number of Qa-2 positive granulocytes in PBMC was lower in BD mice than in BD asymptomatic or normal healthy mice. Among BD mice, the Qa-2 frequency of PBMC in BD eye mice was lower than in BD skin mice, and the differences were larger in granulocytes than lymphocytes. mRNA expression also showed a pattern similar to the FACS frequency. Furthermore, we found that the *in vivo *injection of Qa-2 siRNA reduced the Qa-2 mRNA and protein levels in PBMC of BD mice and deteriorated BD symptoms. Taken together, these findings indicate that down-regulation of Qa-2 could be an important factor in worsening of BD symptoms.

It has been reported that genetic variants with a 14-bp deletion polymorphism in the HLA-G region are associated with Kawasaki disease [34], juvenile idiopathic arthritis [35], ulcerative colitis, and Crohn's disease [36]. In patients with Behcet's disease, the frequency of haplotypes containing the HLA-G *3741_3754 *14 base pair insertion and 1597*delC was found to increase, and this insertion was associated with a lower serum level of HLA-G [26]. In the present study, we found that Qa-2 mRNA and Qa-2 positive PBMCs were significantly lower in BD symptomatic mice than in normal healthy mice.

RNA interference has emerged as a powerful tool to inhibit protein expression [37], and we previously reported that TNF alpha siRNA and IL-6 siRNA inhibited the serum protein level of TNF alpha and IL-6 *in vivo *in the BD mouse model [38,39]. In the present study, Qa-2 siRNA was found to reduce Qa-2 mRNA levels and protein expression *in vitro *in PBMCs isolated from normal mice, and intravenous injection of siRNA into BD mice down-regulated the frequency of Qa-2 expression in lymphocytes and granulocytes of BD mice. Treatment of BD mice with Qa-2 siRNA resulted in deterioration of symptoms such as skin ulcer and arthritis, and decreased Qa-2 levels were found to be related to changes in the disease progression. Control siRNA injection to BD mice did not change the BD symptoms and disease severity score. The inhibitory function of HLA-G might be important in regulation of the immune responses [40].

HLA-G also influences the Th cytokine balance toward Th2 by promoting the secretion of IL-3, IL-4 and IL-10 while down-regulating the production of IFNγ and TNFα [41-43]. In the present study, Qa-2 siRNA increased the IFNγ mRNA levels in the spleens of BD mice, whereas control siRNA did not increase the IFNγ mRNA levels. The increase in IFNγ mRNA levels after injection of Qa-2 siRNA to BD mice was not due to a non-specific immune response, but rather to down-regulation of Qa-2. In addition, the present results showed that the injection of Qa-2 expression vector decreased IFNγ-stained macrophages in BD mice.

It has been suggested that genetic, immunologic and inflammatory factors play a significant role in susceptibility to BD [44]. NK cells play a role in induction and/or regulation of various types of immune responses, including several autoimmune diseases, through cytotoxicity and cytokine production [45]. Several studies have shown natural killer (NK)-mediated cytotoxicity, and cytokine secretion is believed to play roles in the immunopathogenesis of Behcet's disease [46,47]. Functionally, HLA-G directly inhibits the cytolytic function of peripheral blood NK cells [48]. The frequency of NK cells was found to be higher in BD mice than BDN mice. Increased numbers of NK cells have been reported in patients with BD [49]. The down-regulation of Qa-2 by siRNA might increase the number of NK cells, and the increase of NK cells might play an important role in the pathogenesis of BD.

## Conclusions

Qa-2 levels were lower in the PBMC of BD mice than in the PBMC of normal mice. In addition, Qa-2 levels were lower in BD mice with eye involvement than in BD mice with mucocutaneous involvement and BD asymptomatic mice. Qa-2 siRNA effectively reduced Qa-2 mRNA expression in PBMC culture and the frequency of Qa-2 positive PBMC in BD mice, indicating that Qa-2 siRNA effectively reduced Qa-2 expression both *in vitro *and *in vivo*. Qa-2 siRNA was capable of modulating BD-like symptoms, leading to deterioration of BD mice. The results of this study confirmed that decreased Qa-2 levels are related to changes in the disease pattern and deterioration of BD-like symptoms.

## Competing interests

The authors declare that they have no competing interests.

## Authors' contributions

ML conducted the molecular and flow cytometric portions of the study, participated in the *in vivo *experiment, and drafted the manuscript. BC participated in the vector construction and *in vitro *experiment. HJK participated in making the BD mouse model. JAS conducted the experiments on the NK cells. KSP and ESL participated in the design of the study and discussion of data analysis. SS conceived of the study, participated in its design and coordination, and helped draft the manuscript. All authors read and approved the final manuscript.

## References

[B1] Geraghty DE Koller BH Orr HT A human major histocompatibility complex class I gene that encodes a protein with a shortened cytoplasmic segment Proc Natl Acad Sci USA 1987 84 9145 9149 10.1073/pnas.84.24.9145 3480534 PMC299709

[B2] Ellis SA Sargent IL Redman CW McMichael AJ Evidence for a novel HLA antigen found on human extravillous trophoblast and a choriocarcinoma cell line Immunology 1986 59 595 601 3804380 PMC1453327

[B3] Crisa L McMaster MT Ishii JK Fisher SJ Salomon DR Identification of a thymic epithelial cell subset sharing expression of the class Ib HLA-G molecule with fetal trophoblasts J Exp Med 1997 186 289 298 10.1084/jem.186.2.289 9221758 PMC2198976

[B4] Torres MI Lopez-Casado MA Luque J Rios A New advances in celiac disease: serum and intestinal expression of HLA-G Int Immunol 2006 18 713 718 10.1093/intimm/dxl008 16569678

[B5] Rizzo R Hviid TV Stignani M Balboni A Grappa MT Melchiorri L Baricordi OR The HLA-genotype is associated with IL-10 levels in activated PBMCs Immunogenetics 2005 57 172 181 10.1007/s00251-005-0788-0 15900488

[B6] Carosella ED Favier B Rouas-Freiss N Moreau P Lemaoult J Beyond the increasing complexity of the immunomodulatory HLA-G molecule Blood 2008 111 4862 70 10.1182/blood-2007-12-127662 18334671

[B7] Riteau B Rouas-Freiss N Menier C Paul P Dausset J Carosella ED HLA-G2, -G3 and -G4 isoforms expressed as nonmature cell-surface glycoproteins inhibit NK and antigen-specific CTL cytolysis J Immunol 2001 166 5018 5026 11290782 10.4049/jimmunol.166.8.5018

[B8] Rajagopalan S Long EO A human histocompatibility leukocyte antigen (HLA)-G-specific receptor expressed on all natural killer cells J Exp Med 1999 189 1093 1099 10.1084/jem.189.7.1093 10190900 PMC2193010

[B9] Dorling A Monk NJ Lechler RI HLA-G inhibits the transendothelial migration of human NK cells Eur J Immunol 2000 30 586 593 10.1002/1521-4141(200002)30:2<586::AID-IMMU586>3.0.CO;2-Y 10671215

[B10] Kanai T Fujii T Unno N Yamashita T Hyodo H Miki A Hamai Y Kozuma S Taketani Y Human leukocyte antigen-G-expressing cells differently modulate the release of cytokines from mononuclear cells present in the decidua versus peripheral blood Am J Reprod Immunol 2001 45 94 99 10.1111/j.8755-8920.2001.450205.x 11216880

[B11] Riteau B Menier C Khalil-Daher I Sedlik C Dausset J Rouas-Freiss N Carosella ED HLA-G inhibits the allogeneic proliferative response J Reprod Immunol 1999 43 203 211 10.1016/S0165-0378(99)00034-0 10479056

[B12] Bainbridge DR Ellis SA Sargent IL HLA-G suppresses proliferation of CD4+ T lymphocytes J Reprod Immunol 2000 48 17 26 10.1016/S0165-0378(00)00070-X 10996380

[B13] Aractingi S Briand N Le Danff C Viguier M Bachelez H Michel L Dubertret L Carosella ED HLA-G and NK receptor are expressed in psoriatic skin: a possible pathway for regulating infiltrating T cells? Am J Pathol 2001 159 71 77 11438456 10.1016/S0002-9440(10)61675-6 PMC1850403

[B14] Khosrotehrani K Le Danff C Reynaud-Mendel B Dubertret L Carosella ED Aractingi S HLA-G expression in atopic dermatitis J Invest Dermatol 2001 117 750 752 10.1046/j.0022-202x.2001.01487.x 11564188

[B15] Shimizu T Ehrlich GE Inaba G Hayashi K Behcet disease (Behcet syndrome) Semin Arthritis Rheum 1979 8 223 260 10.1016/0049-0172(79)90004-0 382361

[B16] Lee S Bang D Cho YH Lee ES Sohn S Polymerase chain reaction reveals herpes simplex virus DNA in saliva of patients with Behçet's disease Arch Dermatol Res 1996 288 179 183 10.1007/BF02505221 8967789

[B17] Lee ES Lee S Bang D Hamza M Herpes simplex virus detection by polymerase chain reaction in intestinal ulcer of patients with Behcet's disease Proceedings of 7th International Conference on Behcet's Disease 1997 Tunis: Pub Adhoua 71 73

[B18] Bang D Cho YH Choi HJ Hamza M Detection of herpes simplex virus DNA by polymerase chain reaction in genital ulcer of patients with Behcet's disease Proceedings of 7th International Conference on Behcet's Disease 1997 Tunis: Pub Adhoua 74 76

[B19] Behcet H Ueber rezidivierende, apthöse, durch ein virus verusachte geschwüre am mund, am auge und an den genitalen Dermatol Wochenschr 1937 36 1152 1157

[B20] Sohn S Lee ES Bang D Lee S Behcet's disease-like symptoms induced by the herpes simplex virus in ICR mice Eur J Dermatol 1998 8 21 23 9649665

[B21] Sohn S Bang D Lee ES Kwon HJ Lee SI Lee S Experimental studies on the antiviral agent famciclovir in Behcet's disease symptoms in ICR mice Br J Dermatol 2001 145 799 804 10.1046/j.1365-2133.2001.04498.x 11736905

[B22] Sohn S Bang D Lee SI Kim YA Lee ES Ha JY Kim JH Choi SY Lee S Combined treatment with colchicine and Herba Taraxaci (Tarazacum mongolicum Hand.-Mazz.) attenuates Behcet's disease-like symptoms in mice and influences the expressions of cytokines Int Immunopharmacol 2003 3 713 721 10.1016/S1567-5769(03)00071-7 12757740

[B23] Lee ES Kim YA Kwon HJ Bang D Lee S Sohn S Thalidomide up-regulates macrophage inflammatory protein-1 in herpes simplex virus-induced Behcet's disease-like animal model Arch Derm Res 2004 296 175 181 15290170 10.1007/s00403-004-0498-8

[B24] Frassanito MA Dammacco R Cafforio P Dammacco F Th1 polarization of the immune response in Behcet's disease: a putative pathogenetic role of interleukin-12 Arthritis Rheum 1999 42 1967 1974 10.1002/1529-0131(199909)42:9<1967::AID-ANR24>3.0.CO;2-Z 10513814

[B25] Sohn S Lee ES Kwon HJ Lee SI Bang D Lee S Expression of Th2 cytokines decreases the development of and improves Behçet's disease-like symptoms induced by herpes simplex virus in mice J Infect Dis 2001 15 1180 1186 10.1086/319681 11262199

[B26] Park KS Nam JH Lee ES Choi JS Bang D Lee S Increased risk of human leukocyte antigen-G gene variants in Behçet's disease Clin Exp Rheumatol 2006 24 S126 S127 Erratum in: Clin Exp Rheumatol. 2007, 25:507-508 17067446

[B27] Park KS Park JS Nam JH Bang D Sohn S Lee ES HLA-E*0101 and HLA-G*010101 reduce the risk of Behcet's disease Tissue Antigens 2007 69 139 144 10.1111/j.1399-0039.2006.00742.x 17257316

[B28] Ulker N Lewis KD Hood LE Stroynowski I Activated T cells transcribe an alternatively spliced mRNA encoding a soluble form of Qa-2 antigen EMBO J 1990 9 3839 4387 2249652 10.1002/j.1460-2075.1990.tb07602.x PMC552151

[B29] Jackson AL Bartz SR Schelter J Kobayashi SV Burchard J Mao M Li B Cavet G Linsley PS Expression profiling reveals off-target gene regulation by RNAi Nat Biotechnol 2003 21 635 637 10.1038/nbt831 12754523

[B30] Sledz CA Holko M de Veer MJ Silverman RH Williams BR RNA interference and double-stranded-RNA-activated pathways Biochem Soc Trans 2004 32 952 956 10.1042/BST0320952 15506933

[B31] Hamar P Song E Kokeny G Chen A Ouyang N Lieberman J Small interfering RNA targeting Fas protects mice against renal ischemia-reperfusion injury Proc Natl Acad Sci USA 2004 101 14883 14888 10.1073/pnas.0406421101 15466709 PMC522049

[B32] Xie FY Woodle MC Lu PY Harnessing in vivo siRNA delivery for drug discovery and therapeutic development Drug Discov Today 2006 11 67 73 10.1016/S1359-6446(05)03668-8 16478693 PMC7108327

[B33] Rieger L Hofmeister V Probe C Dietl J Weiss EH Steck T Kämmerer U Th1- and Th2-like cytokine production by first trimester decidual large granular lymphocytes is influenced by HLA-G and HLA-E Mol Hum Reprod 2002 8 255 261 10.1093/molehr/8.3.255 11870233

[B34] Kim JJ Hong SJ Hong YM Kim S Kang MJ Kim KJ Seo EJ Yoo HW Cheong HS Shin HD Park IS Lee JK Genetic variants in the HLA-G region are associated with Kawasaki disease Hum Immunol 2008 69 867 871 10.1016/j.humimm.2008.10.002 18976687

[B35] Veit TD Vianna P Scheibel I Brenol CV Brenol JC Xavier RM Delgado-Cañedo A Gutierrez JE Brandalize AP Schuler-Faccini L Chies JA Association of the HLA-G 14-bp insertion/deletion polymorphism with juvenile idiopathic arthritis and rheumatoid arthritis Tissue Antigens 2008 71 440 446 10.1111/j.1399-0039.2008.01019.x 18331529

[B36] Glas J Török HP Tonenchi L Wetzke M Beynon V Teshome MY Cotofana S Schiemann U Griga T Klein W Epplen JT Folwaczny C Folwaczny M Mussack T Weiss EH The 14-bp deletion polymorphism in the HLA-G gene displays significant differences between ulcerative colitis and Crohn's disease and is associated with ileocecal resection in Crohn's disease Int Immunol 2007 19 621 626 10.1093/intimm/dxm027 17446213

[B37] Pawar RM Raj GD Kumar TM Raja A Balachandran C Effect of siRNA mediated suppression of signaling lymphocyte activation molecule on replication of peste des petits ruminants virus in vitro VirusResearch 2008 136 118 123 10.1016/j.virusres.2008.04.026 PMC7127705 18550191

[B38] Choi B Hwang Y Kwon HJ Lee ES Park KS Bang D Lee S Sohn S Tumor necrosis factor alpha small interfering RNA decreases herpes simplex virus-induced inflammation in a mouse model J Dermatol Sci 2008 52 87 97 10.1016/j.jdermsci.2008.05.001 18585901

[B39] Shim J Byun HO Lee YD Lee ES Sohn S Interleukin-6 small interfering RNA improved the herpes simplex virus-induced systemic inflammation in vivo Behcet's disease-like mouse model Gene Ther 2009 16 415 425 10.1038/gt.2008.180 19092856

[B40] Trowsdale J Betz AG Mother's little helpers: mechanisms of maternal-fetal tolerance Nat Immunol 2006 7 241 246 10.1038/ni1317 16482172

[B41] Fujii T Hamai Y Kozuma S Miki A Yamashita T Hyodo H Unno N Taketani Y Effects of sairei-to and tokishakuyaku-san on cytokine release from peripheral blood mononuclear cells upon recognition of HLA-G protein in the treatment of recurrent abortion Methods Find Exp Clin Pharmacol 1999 21 261 264 10.1358/mf.1999.21.4.538174 10399132

[B42] Rieger L Hofmeister V Probe C Dietl J Weiss EH Steck T Kämmerer U Th1- and Th2-like cytokine production by first trimester decidual large granular lymphocytes is influenced by HLA-G and HLA-E Mol Hum Reprod 2002 8 255 261 10.1093/molehr/8.3.255 11870233

[B43] Carosella ED Moreau P Aractingi S Rouas-Freiss N HLA-G: a shield against inflammatory aggression Trends Immunol 2001 22 553 555 10.1016/S1471-4906(01)02007-5 11574278

[B44] Zierhut M Mizuki N Ohno S Inoko H Gül A Onoé K Isogai E Immunology and functional genomics of Behçet's disease Cell Mol Life Sci 2003 60 1903 1922 10.1007/s00018-003-2333-3 14523551 PMC11138769

[B45] Carnaud C Lee D Donnars O Park SH Beavis A Koezuka Y Bendelac A Cross-talk between cells of the innate immune system: NKT cells rapidly activate NK cells J Immunol 1999 163 4647 4650 10528160

[B46] Ahn JK Chung H Lee DS Yu YS Yu HG CD8brightCD56+ T cells are cytotoxic effectors in patients with active Behcet's uveitis J Immunol 2005 175 6133 6142 16237110 10.4049/jimmunol.175.9.6133

[B47] Takeno M Shimoyama Y Kashiwakura J Nagafuchi H Sakane T Suzuki N Abnormal killer inhibitory receptor expression on natural killer cells in patients with Behçet's disease Rheumatol Int 2004 24 212 216 10.1007/s00296-003-0352-x 12879269

[B48] Rouas-Freiss N Marchal RE Kirszenbaum M Dausset J Carosella ED The alpha1 domain of HLA-G1 and HLA-G2 inhibits cytotoxicity induced by natural killer cells: Is HLA-G the public ligand for natural killer cell inhibitory receptors? Proc Natl Acad Sci USA 1997 94 5249 10.1073/pnas.94.10.5249 9144223 PMC24664

[B49] Kaneko F Takahashi Y Muramatsu R Adachi K Miura Y Nakane A Minagawa T Natural killer cell numbers and function in peripheral lymphoid cells in Behcet's disease Br J Dermatol 1985 113 313 318 10.1111/j.1365-2133.1985.tb02083.x 4063167

